# Testing proposed quality measures for treatment of carpal tunnel syndrome: feasibility, magnitude of quality gaps, and reliability

**DOI:** 10.1186/s12913-020-05704-6

**Published:** 2020-09-11

**Authors:** Alex H. S. Harris, Esther L. Meerwijk, Qian Ding, Amber W. Trickey, Andrea K. Finlay, Eric M. Schmidt, Catherine M. Curtin, Erika D. Sears, Teryl K. Nuckols, Robin N. Kamal

**Affiliations:** 1 Center for Innovation to Implementation, VA Palo Alto Healthcare System, Palo Alto, CA USA; 2 Department of Surgery, Stanford–Surgery Policy Improvement Research and Education Center, Stanford, CA USA; 3 Michigan Medicine Department of Surgery, Center for Clinical Management Research, VA Ann Arbor Health Care System, Ann Arbor, MI USA; 4 Cedars Sinai Medical Center, 8700 Beverly Blvd, Los Angeles, CA USA; 5 Department of Orthopedic Surgery, Stanford University School of Medicine, Stanford, CA USA

**Keywords:** Quality measurement, Hand surgery, Carpal tunnel syndrome, Veterans, carpal tunnel release

## Abstract

**Background:**

The American Academy of Orthopaedic Surgeons and American Society for Surgery of the Hand recently proposed three quality measures for carpal tunnel syndrome (CTS): Measure 1 - Discouraging routine use of Magnetic resonance imaging (MRI) for diagnosis of CTS; Measure 2 - Discouraging the use of adjunctive surgical procedures during carpal tunnel release (CTR); and Measure 3 - Discouraging the routine use of occupational and/or physical therapy after CTR. The goal of this study were to 1) Assess the feasibility of using the specifications to calculate the measures in real-world healthcare data and identify aspects of the specifications that might be clarified or improved; 2) Determine if the measures identify important variation in treatment quality that justifies expending resources for their further development and implementation; 3) Assess the facility- and surgeon-level reliability of measures.

**Methods:**

The measures were calculated using national data from the Veterans Health Administration (VA) Corporate Data Warehouse for three fiscal years (FY; 2016–18). Facility- and surgeon-level performance and reliability were examined. To expand the testing context, the measures were also tested using data from an academic medical center.

**Results:**

The denominator of Measure 1 was 132,049 VA patients newly diagnosed with CTS. The denominators of Measures 2 and 3 were 20,813 CTRs received by VA patients. The median facility-level performances on the three measures were 96.5, 100, and 94.7%, respectively. Of 130 VA facilities, none had < 90% performance on Measure 1. Among 111 facilities that performed CTRs, only 1 facility had < 90% performance on Measure 2. In contrast, 21 facilities (18.9%) and 333 surgeons (17.8%) had lower than 90% performance on Measure 3 (Median facility- and surgeon-level reliability for Measure 3 were very high (0.95 and 0.96 respectively).

**Conclusions:**

Measure 3 displayed adequate facility- and surgeon-level variability and reliability to justify its use for quality monitoring and improvement purposes. Measures 1 and 2 lacked quality gaps, suggesting they should not be implemented in VA and need to be tested in other healthcare settings. Opportunities exist to refine the specifications of Measure 3 to ensure that different organizations calculate the measure in the same way.

## Background

Carpal tunnel syndrome (CTS) involves compression of the median nerve at the wrist and can cause debilitating symptoms, such as pain, numbness or tingling in the fingers, loss of sleep, and thumb weakness [1]. Among employed adults in the United States (US), the prevalence of CTS has been estimated to be 7.8%, the incidence rate to be 2.3 per 100 person-years, and the total associated medical costs of $2 billion [2, 3]. In addition to non-operative treatments provided in diverse healthcare settings, surgeons in the US annually complete more than 500,000 carpal tunnel releases (CTRs) to treat this common and disabling syndrome [4].

In 2016, the American Academy of Orthopaedic Surgeons (AAOS) published the “Management of Carpal Tunnel Syndrome Evidence-Based Clinical Practice Guideline” [1], also endorsed by the American Society for Surgery of the Hand (ASSH) and the American College of Radiology. The multi-disciplinary guideline panel examined dozens of diagnostic and treatment practices for CTS and used a rigorous and systematic process to evaluate the strength of evidence linking each practice to important clinical outcomes. These guidelines represent a broad-based consensus regarding practice standards for the diagnosis and treatment of CTS. However, for evidence-graded clinical practice guidelines to improve the quality of care, practice recommendations with strong evidentiary support need to be operationalized into feasible, reliable, and valid healthcare quality measures [5]. When healthcare quality measures are developed, pilot tested, validated, and implemented to monitor consensus practice standards, then clinicians, administrators, policy makers, and patients can use them for diverse purposes [6].

Recognizing the need to operationalize consensus standards of care for CTS into feasible and valid quality measures, the ASSH/AAOS Carpal Tunnel Quality Measures Workgroup was convened in January 2017 to review the clinical practice guidelines with the purpose of selecting quality concepts and drafting initial specifications of process-oriented quality measures [7]. The workgroup engaged in a modified Delphi process to judge 22 quality concepts with strong or moderate supporting evidence (3–4 stars on a 1–4 star scale) from the clinical practice guideline. Strong evidence (4 stars) was defined as “Evidence from two or more “High” quality studies with consistent findings for recommending for or against the intervention” [1]. Moderate evidence (3 Stars) was defined as “Evidence from two or more “Moderate” quality studies with consistent findings, or evidence from a single “High” quality study for recommending for or against the intervention” [1]. The workgroup then evaluated the quality concepts in terms of the National Quality Forum (NQF) criteria of importance, acceptability, feasibility, and usability. Most of the quality concepts were deemed infeasible to operationalize with commonly available data. More details of the workgroup’s process and results are in a publicly available technical report [7].

Table 1 presents the 3 measures that were selected by the workgroup for development and specification [7]. Measure 1 (“Avoidance of MRIs for diagnosis of CTS”) is motivated by evidence that MRIs are a poor rule-out test for CTS as compared to clinical examination or nerve conduction studies [8]. Measure 2 (“Avoidance of Adjunctive Procedures during CTR”) is motivated by studies failing to find benefits of adjunctive procedures (i.e., internal neurolysis with operating microscope, radical nine-tendon flexor synovectomy, tenolysis) beyond the benefits of CTR alone [9–15]. Measure 3 (“Avoidance of Routine in-clinic OT/PT after CTR”) is motivated by studies failing to find benefits of in-clinic occupational and/or physical therapy (OT/PT) modalities after CTR compared to home programs or placebo [16–19].

**Table 1:** Quality Concepts Selected by the ASSH/AAOS Workgroup for Development and Pilot Testing [7]

**Measure 1**: **Discouraging routine use of MRI for diagnosis of CTS** **Description**: Percent of patients diagnosed with CTS not receiving an MRI in the 90 days before or after the diagnosis.	
**Type**: Process measure using claims data	
**Denominator**: Patients with a diagnosis of CTS (ICD-10-CM: G56.00, G56.01, G56.02, G56.03^a^ or ICD-9-CM: 354.0)	
**Numerator**: Patients with a diagnosis of CTS who did not receive an ipsilateral wrist MRI to evaluate for carpal tunnel syndrome within 90 days before or after the diagnosis (CPT: 73218, 73219, 73220, 73221, 73222, 73223)	
**Strength of evidence from CPG:** Moderate	
**Rationale:** MRIs are a weak or poor a rule out test for CTS as compared to hand pain diagrams and nerve conduction studies [8].	
**Measure 2: Discouraging adjunctive surgical procedures during carpal tunnel release (CTR)**	
**Description**: Percent of patients diagnosed with CTS who receive CTR who did not receive the following procedures at the same time: Internal neurolysis using operating microscope, radical nine-tendon flexor synovectomy, tenolysis of flexor or extensor tendon, forearm and/or wrist. **Type**: Process measure using claims data.	
**Denominator**: Patients with a diagnosis of CTS (ICD-10-CM: G56.00, G56.01, G56.02, G56.03^a^ or ICD-9-CM: 354.0) who receive CTR (CPT: 64721 or 29848).	
**Numerator**: Patients who did not have any one of the listed procedures completed at the same time (CPT 64727, 25115, 25295).	
**Strength of evidence from CPG:** Moderate	
**Rationale:** Studies have failed to find benefits of adjunctive procedures beyond those for CTR alone [9–15].	
**Measure 3. Discouraging routine use of in-clinic occupational and/or physical therapy after CTR**	
**Description**: Percent of patients who received CTR and were not prescribed in-clinic postoperative hand, physical, or occupational therapy within 6 weeks after release.	
**Type**: Process measure using claims data.	
**Denominator**: Patients with a diagnosis of CTS (ICD-10-CM: G56.00, G56.01, G56.02, G56.03^a^ or ICD-9-CM: 354.0) who receive CTR (CPT: 64721 or 29,848)	
**Numerator**: Patients who did not receive in-clinic postoperative hand physical therapy (low, moderate, or high complexity) or occupational therapy (low, moderate, or high complexity) within 6 weeks of CTR (CPT 97161, 97162, 97163, 97165, 97166, 97167)	
**Strength of evidence from CPG:** Moderate	
**Rationale:** Moderate quality studies have failed to find benefits to CTR postoperative rehabilitation of in-clinic OT/PT modalities compared to home programs or placebo [16–19].	

^a^ Included in our calculation but not included in most recent technical report [4].

Once quality measures have been proposed and initial specifications have been developed, it is essential to pilot test them in real-world healthcare data at the intended levels of measurement (e.g., surgeons, facilities, healthcare systems). Pilot testing of quality measures has several goals including: (a) determining the feasibility of using the measure specifications and available data elements to calculate the measures in healthcare data from diverse settings, and relatedly, identifying aspects of the specification that might be improved or clarified [20]; (b) assessing if the distribution of quality measure performance is varied enough to justify measure implementation, such that sufficient opportunity for improvement exists; c) examining the measures’ reliability – meaning their ability to distinguish real differences in performance between measurement units (aka signal-to-noise-ratio) [21].

Initial specification and pilot testing of Measures 1–3 was done in 2012–2014 Truven Marketscan data which precluded testing of International Classification of Diseases, Tenth Revision, Clinical Modification (ICD-10-CM) versions of the measures, and did not include facility or surgeon identifiers, making it impossible to assess if meaningful variation exists between the units to which the measures will be applied (i.e., surgeons and healthcare facilities) [7].

The goal of this study was to test if the three CTS quality measures selected by ASSH/AAOS Carpal Tunnel Quality Measures Workgroup might be useful for monitoring and improving the quality of CTS treatment. We conducted measure testing in data from the Veterans Health Administration (VA), the largest publicly-funded integrated healthcare system in the US, and secondarily in a private academic medical center. Specifically, we sought to 1) Identify aspects of the specifications that might be clarified or improved; 2) Determine if the measures identify variation in treatment quality that justifies expending resources for their further development and implementation; 3) Assess the facility- and surgeon-level reliability of measures.

## Methods

### Study design and setting

All three measures were operationalized according to the AAOS/ASSH specifications [7] (Table 1) using national data from the Veterans Health Administration (VA) Corporate Data Warehouse (CDW) for each of three fiscal years (FY; 2016–18), and as one three-year measurement period. The VA CDW contains all clinically and administratively generated data (e.g., encounter, procedure, diagnosis data) from all VA facilities nationally. Measure data were aggregated to the level of major VA medical facilities. Overall performance and facility-level variation in each measure’s performance was assessed. The distribution of surgeon-level performance was also evaluated for Measures 2 and 3, which are focused on carpal tunnel release (CTR) surgery. Although no established standards exist regarding what constitutes an adequate quality gap and variability to justify implementation of quality measures, we describe the proportion of measurement units (i.e., facilities, surgeons) who fall below 90% performance. If all, or almost all, measurement units have better than 90% performance, we consider the quality gap, and opportunity for further quality improvement, to be low.

For measures with adequate variability, facility- and surgeon-level reliability analysis was conducted. Reliability in this context refers to how well a measure distinguishes real differences in quality between measurement units (signal) in the presence of measurement error (noise), and is characterized with beta-binomial signal-to-noise ratio [21]. This is the standard measure of quality measure reliability when accountable entities, VA facilities and surgeons in this case, are being measured on the proportion of patients that meet some standard [20, 21]. This signal-to-noise ratio ranges between 0 when all of the variability can be attributed to measurement error and 1 when all the variability is due to real differences in quality. In addition to calculating overall reliability, we examined the impact of various minimum case numbers (e.g., 5 vs. 30 over the measurement period) on reliability estimates.

In order to test the measures in a different healthcare context, they were also calculated for 2014–2016 Stanford Health Care (SHC) data using the STAnford medicine Research data Repository (STARR), a clinical data warehouse containing live electronic medical records, including all diagnoses, procedures, and encounter-level patient data. Overall measure performance was calculated, but SHC surgeon identifiers were not available.

### Measures

#### Measure 1 (avoidance of MRIs for diagnosis of CTS)

As summarized in Table 1, the denominator of the measure is all patients with a diagnosis of CTS during the measurement period. We added G56.03 – bilateral CTS – to the denominator definition and suggest the technical manual should also address this omission. Although not clearly specified in the technical manual, we chose each patient’s first diagnosis of CTS in the measurement period as the index encounter. The CTS diagnoses were included regardless of the order of diagnoses recorded for the encounter. The numerator was the number of patients who did not receive an upper extremity MRI in the 90 days before or after the index encounter.

#### Measure 2 (avoidance of adjunctive procedures during CTR)

The denominator of the measure is all patients receiving CTR during the measurement period. The numerator was the number of patients who did not receive at least one of the adjunctive treatments at the same time.

#### Measure 3 (avoidance of routine in-clinic OT/PT after CTR)

The denominator of the measure is all patients receiving CTR during the measurement period. The numerator was the number of patients who did not receive in-clinic postoperative hand physical therapy or occupational therapy (OT/PT) within 6 weeks of CTR. To address concerns that the OT/PT visits were for something unrelated to CTS or CTR, we also pilot tested a version of Measure 3 that required that the OT/PT visit include a CTS diagnosis.

## Results

### Measure 1

Of 132,049 VA patients who were diagnosed with CTS in FY16–18, 5066 (3.8%) received an upper extremity MRI (nationwide Measure 1 performance = 96.2%). Performance on Measure 1 across 130 VA facilities ranged from 90 to 100% (Table 2). The results for each year from FY16 to FY18 were very similar. At SHC, only 1.9% percent of 4304 patient diagnosed with CTS received an upper extremity MRI (Measure 1 performance = 98.1%).

**Table 2:** Measure 1**:** Discouraging routine use of MRI for diagnosis of CTS

System	Time	Denominator	Numerator	Performance	Facility-level Range
VA	2016	44,302	42,627	96.2	88.0–100.0
VA	2017	42,897	41,253	96.2	89.9–100.0
VA	2018	44,850	43,083	96.1	88.4–100
VA	FY16–18	132,049	126,983	96.2	90.0–100.0
SHC	2014–16	4298	4221	98.1%	NA

#### Possible improvements and clarifications to the technical specifications

The technical manual states “This measure is to be reported at each denominator eligible visit occurring during the reporting period for patients with a diagnosis of carpal tunnel syndrome who are seen during the reporting period.” The definition of “denominator eligible visit” is unclear. We assumed that each patient should have only one denominator eligible visit (“index encounter”) per measurement period, corresponding to their first CTS diagnosis. If the intention is to focus on the initial diagnosis of CTS, then a diagnosis-free period before the index encounter should be required. The ICD-10-CM code G56.03 (Carpal tunnel syndrome, bilateral upper limbs) should be included in the specifications. Also, as some VA patients were diagnosed with CTS initially as inpatients, the specification should be specific about whether inpatient diagnoses should be included.

### Measure 2

Of 20,813 VA patients who received carpal tunnel release in FY16–18, only 31 received an adjunctive procedure (nationwide Measure 2 performance = 99.9%; Table 3). Among 111 facilities and 1873 surgeons that performed CTRs, 1 facility and 10 surgeons had lower than 90% performance on Measure 2. The results for each year from FY16 to FY18 were very similar. In SHC, only 21 of the 640 patients receiving CTR had an adjunctive procedure (Measure 2 performance = 96.7%).

**Table 3:** Measure 2**:** Discouraging adjunct procedures during CTR

System	Time	Denominator	Numerator	Performance	Facility-level Range
VA	2016	7530	7517	99.8	72.7–100.0
VA	2017	7070	7059	99.8	95.2–100.0
VA	2018	6213	6206	99.9	88.9–100.0
VA	FY16–18	20,813	20,782	99.9	72.7–100.0
SHC	2014–16	640	619	96.7%	NA

#### Possible improvements and clarifications to the technical specifications

The technical manual states the denominator should be the “number of patients who underwent carpal tunnel release” and also that “This measure is to be reported at each denominator eligible visit occurring during the reporting period.” Therefore, it is unclear if the denominator should be limited to one (first?) CTR per patient or if all CTRs should be included. For pilot testing, we included the first CTR per patient in each reporting period. It also important to note that measure statement is intended to discourage “internal neurolysis using operating microscope”, but code 64727 is not that specific: “Neuroplasty (Exploration, Neurolysis or Nerve Decompression) Procedures on the Extracranial Nerves, Peripheral Nerves, and Autonomic Nervous System”. Similarly, the measure statement discourages “radical nine-tendon flexor synovectomy” but code 25115 is less specific “Radical excision of bursa, synovia of wrist, or forearm tendon sheaths (eg, tenosynovitis, fungus, Tbc, or other granulomas, rheumatoid arthritis)”. Therefore, the measures are capturing a somewhat broader range of procedures than suggested by the measure description.

### Measure 3

**Fig. 1:**
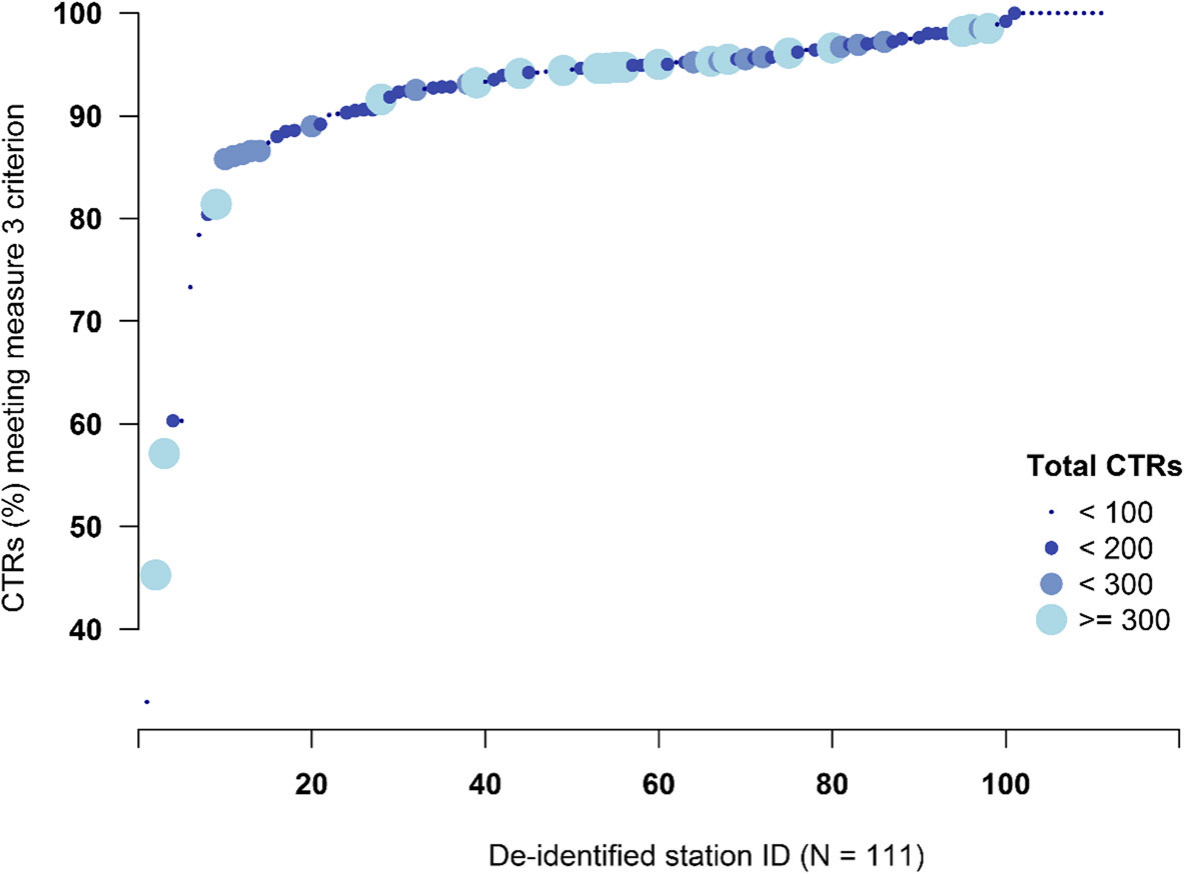
Distribution of Facility-Level Performance on Measure 3: Percent of CTR Patients Not Receiving OT/PT in the 6 Postoperative Weeks. The x-axis represents unique VA facilities, so each dot represents the performance of a different facility. The facilities have been sorted by performance so the distribution is more visually interpretable

**Fig. 2:**
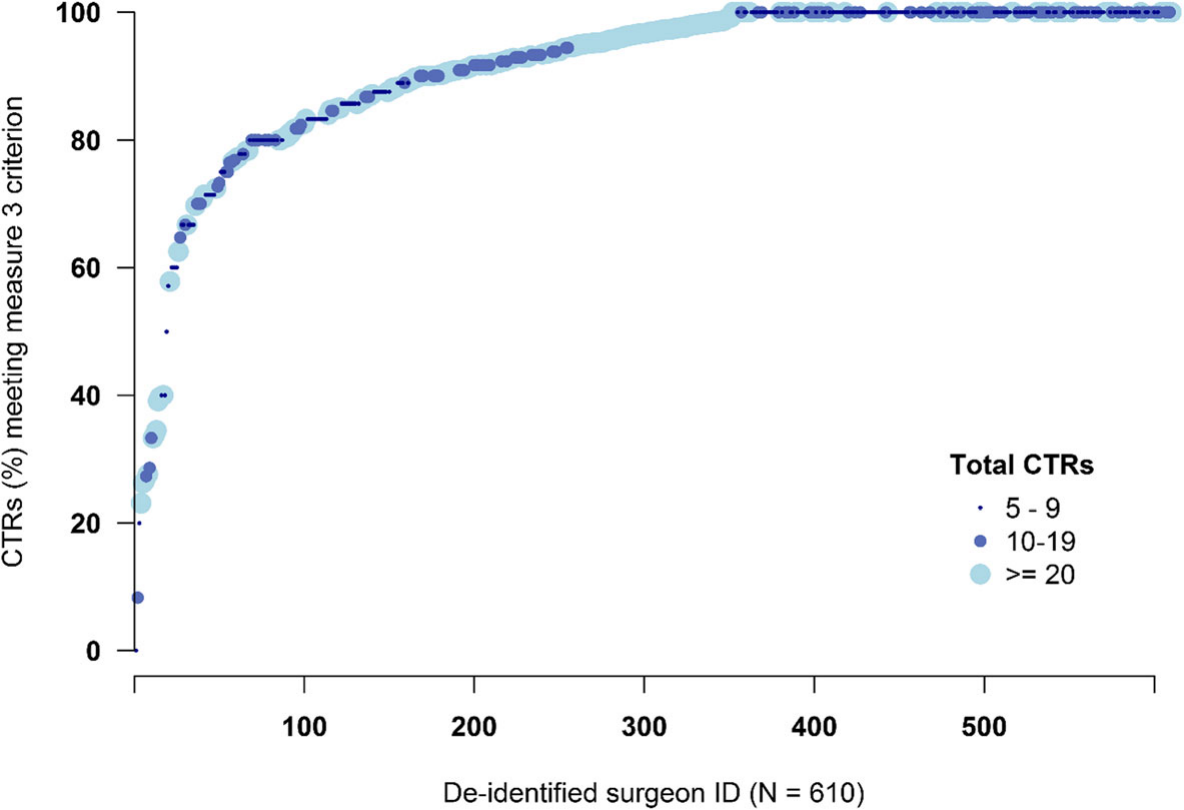
Distribution of Surgeon-Level (CTR > 5) Performance on Measure 3: Percent of CTR Patients Not Receiving OT/PT in the 6 Postoperative Weeks. The x-axis represents unique VA surgeons, so each dot represents the performance of a different surgeon. The surgeons have been sorted by performance so the distribution is more visually interpretable

Of 20,813 VA patients who received carpal tunnel release, 1814 patients received in-clinic OT/PT in the postoperative 6 weeks after CTR (nationwide Measure 3 performance = 91.3%; Table 4). Twenty-one (21) facilities (18.9%) had lower than 90% performance (min-max 32.9–100%) on Measure 3 (Fig. 1), and 333 surgeons (17.8%) had lower than 90% performance. Restricting the analyses to 610 surgeons who performed at least 5 CTR, 179 (29.4%) had lower than 90% performance on Measure 3 (Fig. 2). The quality measure version that required that the OT/PT visit include a CTS diagnosis resulted in somewhat better performance - 93.5% overall; 13.5% of facilities < 90% performance; 20.6% of surgeons with > 5 CTRs had < 90% performance. In SHC, only 17 of the 640 patients receiving CTR had OT/PT within 6 weeks of their first CTR (Measure 3 performance = 97.3%).

**Table 4:** Measure 3 - Discouraging routine use of in-clinic occupational and/or physical therapy after CTR

System	Time	Denominator	Numerator	Performance	Facility-level Range
VA	2016	7530	7187	95.4%	65.4–100.0
VA	2017	7070	6317	89.3%	14.7–100.0
VA	2018	6213	5495	88.4%	8.0–100.0
VA	FY16–18	20,813	19,455	91.3%	32.9–100.0
SHC	2014–16	640	623	97.3%	NA

The median facility- level reliability for Measure 3 was 0.95 overall (min-max: 0.30–1.00, facility *N* = 111); 0.95 (0.61–1.00, *n* = 105) for facilities with at least 20 cases; and 0.95 (0.62–1.00, *n* = 99) for facilities with at least 50 cases. Median surgeon-level reliability for Measure 3 was 0.99 overall (min-max: 0.08–1.00, surgeon *N* = 1185); 0.96 (0.18–1.00, *n* = 630) for surgeons with at least 5 cases; and 0.95 (0.64–1.00, *n* = 189) for surgeons with at least 30 cases.

#### Possible improvements and clarifications to the technical specifications

The technical manual states “This measure is an inverse measure – lower scores indicate higher quality”. But the numerator definition includes those who do not get OT/PT. Consideration should be given to requiring that the OT/PT encounter includes a CTS diagnosis. Measures 2 and 3 are based on CTRs, but there is no time frame specified for when CTR can happen after CTS diagnosis. For the majority of VA patients in our dataset, CTR occurs within 1 year after diagnosis, but for ~ 20% CTR occurs within 2 years, and ~ 5% after more than 2 years.

## Discussion

For healthcare quality measures to be useful and have potential to drive improvement in care, they need to be clearly specified, feasible to implement with commonly available data at the intended level of aggregation (e.g., facility, surgeon), and reveal a compelling quality gap that needs to be addressed. In evaluating three recently proposed measures of CTS treatment quality in a national publicly-funded healthcare system and a single private academic healthcare system, we found several aspects of the specifications that can be clarified or improved. Revealing opportunities to improve measure specifications is among the most important reasons to test measures in real-world healthcare data. In VA, we also found that the measures were feasible to implement at the facility and surgeon levels. Although we could not obtain surgeon identifiers in SHC data for research purposes, these data would be available for operational and quality monitoring efforts.

Careful pilot testing of quality measures also provides an opportunity to appreciate the inherent tension between accuracy and feasibility when measure developers try to operationalize clinical evidence with billing codes. As noted above, the codes used to capture “internal neurolysis using operating microscope” and “radical nine-tendon flexor synovectomy” capture a somewhat broader range of procedures. Also, because procedure codes do not carry information regarding the indication for the procedure, it is unknown if the MRIs captured by Measure 1 were for CTS diagnosis or something else. While this is a well-known, and to some extent unavoidable, limitation of quality measure design, it is always important to evaluate how much the decisions to enhance feasibility distort the underlying evidence and clinical practice guidelines. Some noise is acceptable as long as it is more or less randomly distributed among accountable entities (e.g., hospitals or surgeons).

Of the three proposed measures, only Measure 3 displayed adequate facility- and surgeon-level variability to justify its use for quality monitoring and improvement purposes, at least in the VA and SHC. The extent to which these results generalize to other settings is unknown. The overall facility- and surgeon-level reliability for Measure 3 was excellent. Limiting the measure to surgeons with some minimum number of cases (e.g., 5 or 30) in the measurement period improved the range of reliability.

The underlying rationale for Measure 3 is to avoid routine use of postoperative in-clinic, instead of unsupervised, OT/PT. As with many quality measures, a baseline rate of OT/PT use is expected and will be warranted in some cases (e.g. CTR in a patient with severe finger arthritis or a patient having substantial postoperative stiffness). A measure’s usefulness comes from identifying facilities or surgeons whose routine practice is suboptimal given the underlying evidence and the performance of their peers. For example, Fig. 1 reveals two high volume facilities that provided OT/PT to almost half of their CTR patients. In addition to future refinements already mentioned, Measure 3 specifications should address rules for handling patients with CTS in conjunction with other hand conditions, CTS affecting both hands, and surgeons with low measure reliability due to very low CTR volume (e.g., < 5 CTRs per year).

At least in the healthcare systems studied here, Measures 1 and 2 did not identify significant quality gaps that need to be addressed, and may not be useful as quality measures. Its good news that these clinical practice guidelines are already being implemented to a high degree. It is possible that testing in other data sources and health care systems would reveal lower performance and/or more variability. These results also suggest that future quality measure workgroups should focus on processes of care that not only have strong evidence and are feasible to specify, but that also have more undesirable variability in practice. The purpose of quality measures is to drive improvement, which presupposes room to improve.

The most important limitation in our evaluation is the reliance on data from only two healthcare systems, making unknown the generalizability of these results. The main reason we supplemented our VA analysis with data from a private academic institution was to explore whether differences exist between public and privately funded systems. While the results were very similar, Stanford is only one institution. The prior testing of these measures in Truven Marketscan data was unable to examine variability at the facility and surgeon levels, however, the overall estimates in performance for Measures 1–3 were 99, 98, and 86% respectively - consistent with our results [7]. Nonetheless, more testing in different contexts may be warranted before reaching final judgements on Measures 1 and 2, or implementing Measure 3. Also, we were forced to make certain decisions where the written specifications were unclear. Although this was a major purpose of the study, it is also possible that handling the ambiguities differently may produce different results.

## Conclusions

We found that Measure 3 (Discouraging the routine use of occupational and/or physical therapy after CTR surgery) displayed adequate facility- and surgeon-level variability and reliability to justify its use for monitoring and quality improvement purposes. Measures 1 and 2 displayed lack of a quality gap, suggesting they should not be implemented unless their importance and measurement characteristics can be demonstrated in other healthcare systems. Opportunities exist to refine the specifications of Measure 3 to ensure different organizations calculate the measure in the same way. The results of this study highlight the importance of testing proposed quality measures in real-world healthcare data before widespread implementation.

## Data Availability

The datasets generated and/or analyzed during the current study were derived from the VA Corporate Data Warehouse and STAnford medicine Research data Repository, and are not publicly available due to identifying nature of patients and providers. However, how data was collected and managed can be shared via the corresponding author on reasonable request.

## Abbreviations

CTS: carpal tunnel syndrome
MRI: magnetic resonance imaging
CTR: carpal tunnel release
VA: Veterans Health Administration
FY: fiscal year
US: United States
AAOS: American Academy of Orthopaedic Surgeons
ASSH: American Society for Surgery of the Hand
NQF: National Quality Forum
OT/PT: occupational and/or physical therapy
ICD-10-CM: International Classification of Diseases, Tenth Revision, Clinical Modification
CDW: Corporate Data Warehouse
STARR: STAnford medicine Research data Repository
SHC: Stanford Health Care

## References

[1] Management of Carpal Tunnel Syndrome Evidence-Based Clinical Practice Guideline [www.aaos.org/ctsguideline.]. Accessed 1 June 2020.

[2] Stapleton MJ (2006). Occupation and carpal tunnel syndrome. ANZ J Surg.

[3] Dale AM, Harris-Adamson C, Rempel D, Gerr F, Hegmann K, Silverstein B, Burt S, Garg A, Kapellusch J, Merlino L (2013). Prevalence and incidence of carpal tunnel syndrome in US working populations: pooled analysis of six prospective studies. Scand J Work Environ Health.

[4] Fajardo M, Kim S, Szabo R (2012). Incidence of carpal tunnel release: trends and implications within the United States ambulatory care setting. J Hand Surg Am.

[5] Donabedian A (2003). An introduction to quality Assurance in Health Care.

[6] National Forum for Health Care Quality Measurement and Reporting: A National Framework for Healthcare Quality Measurement and Reporting. Washington, DC; Author; Am2002.

[7] American Society for Surgery of the Hand and the American Academy of Orthopaedic Surgeons: Management of Carpal Tunnel Syndrome Technical Report. [https://www.aaos.org/uploadedFiles/PreProduction/Quality/Measures/CTS%20Measures%20Technical%20Report.pdf].

[8] Jarvik JG, Yuen E, Haynor DR, Bradley CM, Fulton-Kehoe D, Smith-Weller T, Wu R, Kliot M, Kraft G, Wang L (2002). MR nerve imaging in a prospective cohort of patients with suspected carpal tunnel syndrome. Neurology..

[9] Dias JJ, Bhowal B, Wildin CJ, Thompson JR (2004). Carpal tunnel decompression. Is lengthening of the flexor retinaculum better than simple division?. J Hand Surg Br.

[10] Leinberry CF, Hammond NL, Siegfried JW (1997). The role of epineurotomy in the operative treatment of carpal tunnel syndrome. J Bone Joint Surg Am.

[11] Lowry WE, Follender AB (1988). Interfascicular neurolysis in the severe carpal tunnel syndrome. A prospective, randomized, double-blind, controlled study. Clin Orthop Relat Res.

[12] Mackinnon SE, McCabe S, Murray JF, Szalai JP, Kelly L, Novak C, Kin B, Burke GM (1991). Internal neurolysis fails to improve the results of primary carpal tunnel decompression. J Hand Surg Am.

[13] Blair WF, Goetz DD, Ross MA, Steyers CM, Chang P (1996). Carpal tunnel release with and without epineurotomy: a comparative prospective trial. J Hand Surg Am.

[14] Crnkovic T, Bilic R, Trkulja V, Cesarik M, Gotovac N, Kolundzic R (2012). The effect of epineurotomy on the median nerve volume after the carpal tunnel release: a prospective randomised double-blind controlled trial. Int Orthop.

[15] Shum C, Parisien M, Strauch RJ, Rosenwasser MP (2002). The role of flexor tenosynovectomy in the operative treatment of carpal tunnel syndrome. J Bone Joint Surg Am.

[16] Pomerance J, Fine I (2007). Outcomes of carpal tunnel surgery with and without supervised postoperative therapy. J Hand Surg Am.

[17] Alves Mde P, de Araujo GC (2011). Low-level laser therapy after carpal tunnel release. Rev Bras Ortop.

[18] Fagan DJ, Evans A, Ghandour A, Prabhkaran P, Clay NR (2004). A controlled clinical trial of postoperative hand elevation at home following day-case surgery. J Hand Surg Br.

[19] Provinciali L, Giattini A, Splendiani G, Logullo F (2000). Usefulness of hand rehabilitation after carpal tunnel surgery. Muscle Nerve.

[20] National Quality Forum (2018). Measure Evaluation Criteria and Guidance for Evaluating Measures for Endorsement.

[21] Adams J, Mehrotra A, McGlynn E (2010). Estimating reliability and misclassification in physician profiling: a tutorial.

